# Transition of Lipid Accumulation Product Status and the Risk of Type 2 Diabetes Mellitus in Middle-Aged and Older Chinese: A National Cohort Study

**DOI:** 10.3389/fendo.2021.770200

**Published:** 2021-11-26

**Authors:** Jinyue Yu, Qian Yi, Leying Hou, Ge Chen, Yaojia Shen, Yuan Song, Yimin Zhu, Peige Song

**Affiliations:** ^1^ School of Public Health and Women’s Hospital, Zhejiang University School of Medicine, Hangzhou, China; ^2^ Institute of Child Health, University College London, London, United Kingdom; ^3^ Medical Research and Biometrics Center, National Center for Cardiovascular Diseases, Beijing, China; ^4^ School of Nursing, Henan University of Traditional Chinese Medicine, Zhengzhou, China; ^5^ Department of Epidemiology and Biostatistics, School of Public Health, Zhejiang University School of Medicine, Hangzhou, China

**Keywords:** lipid accumulation product (LAP), type 2 diabetes mellitus, risk factors, transition, China

## Abstract

**Background:**

Lipid accumulation product (LAP), a product of waist circumference (WC) and fasting triglycerides (TG), is a measure of lipid accumulation and an effective predictor of metabolic syndrome. This study aimed to evaluate the associations of LAP and its longitudinal transitions with type 2 diabetes mellitus (T2DM) among middle-aged and older Chinese.

**Methods:**

Data were extracted from the China Health and Retirement Longitudinal Study (2011, 2013, 2015, and 2018). LAP was defined as (WC-65) ×TG for men, and (WC-58) ×TG for women. Participants were classified into high- and low-LAP groups at baseline, and subsequently into four transition patterns during 2011-2015: maintained-high, maintained-low, high-to-low, and low-to-high LAP. The longitudinal transition patterns of LAP on the development of T2DM were assessed by multivariable Cox frailty models.

**Results:**

Overall, 7397 participants were included for analysis, among whom 849 (11.5%) developed T2DM between 2011 and 2018. Women with high-LAP levels at baseline presented a higher risk of T2DM (hazard ratios [HR]=1.37, 95% confidence interval [CI]: 1.07-1.77), while no significant association was found in men. Compared with women with maintained-low LAP pattern, those with transition patterns of low-to-high LAP and maintained-high LAP were at higher risk of T2DM (HR =1.99 and 1.98, both *P*<0.05); however, for men, the significantly positive association was only observed in maintained-high LAP transition pattern (HR=1.53, 95% CI: 1.04-2.23).

**Conclusions:**

Elevated LAP levels and the transition patterns of maintained-high LAP and low-to-high LAP are significant risk factors for T2DM in women. Preventions are needed to combat T2DM at an early dyslipidemic stage.

## Introduction

Diabetes mellitus (DM) is a disease of abnormal glucose metabolism that affects multiple organ systems. Globally, DM has become a high-risk factor for morbidity and mortality (1). According to the International Diabetes Federation, the number of people affected by DM globally in 2019 was 463 million, among whom an estimated 232 million people had undiagnosed DM (2). Type 2 diabetes mellitus (T2DM) accounts for over 90% of patients with DM, bringing profound psychological and physical distress to patients and putting a huge burden on health care systems (3). Evidence shows that the incidence and prevalence of T2DM vary by geographical region, with 79% of patients living in low-to-middle-income countries (2). The overall prevalence of T2DM in China increased sharply from 1.3% in 1980-1989 to 4.5% in 1990-1999, 6.8% in 2000-2009, and 8.7% in 2010-2014. Previous research estimated that by 2025, the prevalence of T2DM in China would increase to 12.5% (4), becoming a serious epidemic that produces considerable socioeconomic pressures on both individuals and the society.

A 2017 Lancet review shows that 60% of patients with T2DM are obese (body-mass index [BMI] ≥30 kg/m²) (3). In China, a population-based survey with 5071 subjects aged 40 years or older in Shanghai demonstrated that obese people had a significantly higher risk of T2DM (5). Apart from obesity, individuals with normal weight but increased visceral adipose tissue content are also characterized by the presence of insulin resistance and impaired glucose tolerance, which are strongly associated with the development of T2DM (4, 6). In the current era when the association between high BMI and the morbidity of T2DM has been well demonstrated, research interests may need to focus more on the measurement of visceral adiposity, especially when visceral adiposity may represent a higher risk of T2DM (7).

Traditional techniques, such as magnetic resonance imaging (MRI) and computed tomography (CT), are currently the gold standard for measuring visceral adiposity (8). However, those are not suitable for routine clinical practice due to high costs, intensive labor, and the hazard posed by the use of radiation. Therefore, an alternative continuous index of visceral adiposity, the lipid accumulation product (LAP), has been proposed. Based on a combination of waist circumference (WC) and fasting triglycerides (TG), LAP could accurately reflect visceral adiposity and be easily obtained (9). A bulk of studies found that compared with conventional obesity indices, such as BMI, waist-to-hip ratio (WHR) and waist-to-height ratio (WHtR), LAP presented a better predictive ability in metabolic syndrome, DM, and impaired fasting glucose (10–12).

Previous studies have reported the efficacy of LAP in identifying visceral adiposity and insulin resistance (8, 13). However, there is a lack of studies investigating the association of LAP and the risk of T2DM among middle-aged and older Chinese (5). Moreover, whether the dynamic transition of LAP across years is associated with the development of T2DM is still poorly understood. Therefore, this study aimed to evaluate the effect of LAP and its transition on the development of T2DM in middle to older aged adults based on a national Chinese cohort study.

## Material and Methods

### Data and Sample

Data were extracted from the China Health and Retirement Longitudinal Study (CHARLS, available at http://charls.pku.edu.cn/en). CHARLS is a national survey in middle to older aged adults in China (aged 45 years and above) that attempts to provide a wealth of information ranging from socio-economic status to health conditions. The survey informs scientific research and priority setting related to the middle-aged and older adult population in China (14, 15).

The national baseline survey of CHARLS was fielded in 2011-2012. A total of 10257 households and 17708 individuals from 150 counties/districts and 450 villages/urban communities were involved across the country. The geographic regions of investigated provinces are presented in **Figure S1** and **Table S1**. The sampling strategy utilized for the CHARLS Survey involved multiple steps. First, all counties/districts were stratified according to region, urban/rural setting and economic conditions, and 150 counties/districts were randomly selected. Then, three primary sampling units (PSU) were randomly selected from each county, namely rural administrative villages and urban communities. Finally, the “CHARLS-GIS” software was used to map at least 24 families within each PSU, in which residents aged 45 years or older were interviewed with his/her spouse through a face-to-face personal interview in June 2011 and were followed up every two years. To date, CHARLS has been conducted for five rounds from 2011 to 2018. Blood tests were conducted in 2011 and 2015.

In total, 17708 participants were successfully interviewed in four rounds of the CHARLS Survey (2011, 2013, 2015 and 2018), with a family response rate of 80.5%. Individuals who were aged ≥ 45 years, with complete blood sample and biomarker data, and non-diabetic at baseline were included (n = 9485). After excluding individuals with no follow-up response (n = 531), age < 45 years (n = 194), and incomplete data of LAP at baseline (n = 1363), a total of 7397 subjects were included in the final analysis (**Figure S2**). The demographic, socio-economic, geographical and behavioral characteristics of the included and excluded subjects are outlined in **Table S2**.

This study was approved by the ethics review committee of Peking University and carried out by the National School for Development (China Centre for Economic Research) of Peking University. All participants signed written informed consent.

### Measurements

From 2011 to 2018, trained interviewers collected information on demographics, geographic location, socio-economic status, health-related behaviors, and medical history through structured questionnaires. Anthropometry data were gathered following a standard protocol from the World Health Organization (16). Body weight was measured to the nearest 0.1 kg (in light clothes and without shoes) using a digital scale (Omron, HN-286). Height was measured to the nearest 0.1 cm without shoes on a stadiometer (Seca Corporation, 213). WC was horizontally measured at the middle point of the line between the lower rib and the upper iliac crest without wearing a coat. Blood pressure (BP) was measured at the right arm of the participants using an electronic sphygmomanometer (Omron, hem-7200) at intervals of 45 seconds. Systolic (SBP) and diastolic blood pressures (DBP) were measured three times in the seated position after 10 minutes of rest by use of a sphygmomanometer.

Venous blood samples were collected by professional nurses after fasting for at least 12 hours at night. The whole blood count was performed immediately at the survey sites. The whole blood samples were then stored at 4 degrees Celsius, and the remaining samples were transported to the central Laboratory in Beijing Youanmen Centre for Clinical Laboratory of Capital Medical University for further laboratory analysis (14, 15). The levels of blood glucose, total cholesterol (TC), TG, low density lipoprotein (LDL-C) and high density lipoprotein (HDL-C) were tested by enzyme colorimetry. The Glycosylated hemoglobin (HbA1c) levels were measured by boronated-affinity high-performance liquid chromatography.

### Definitions of Covariates

The tertiles of the natural logarithm of per capita expenditures (ln [PCE]) were used as indicators of family wealth, with the bottom, middle and top tertiles representing poor, middle, and rich status, respectively (17, 18). Participants’ residence was classified into North China, Northeast China, East China, South-central China, Southwest China and Northwest China (see **Figure S1** and **Table S1** for more details). Hypertension was defined by an SBP≥140 mmHg, and/or a DBP≥90 mmHg, and/or a self-reported physician diagnosis, and/or currently with antihypertensive drugs, and/or under other related therapeutic measures (18, 19). Participants were categorized as normal, overweight or obese based on BMI, with cut-off points at 24 kg/m^2^ and 28 kg/m^2^ (20).

### Definitions of LAP and T2DM

LAP was defined as (WC (cm)-65) × (TG concentration (mmol/l)) for men, and (WC (cm)-58) × (TG concentration (mmol/l)) for women (9). This formula included the minimum WC values (65 and 58 cm for men and women, respectively) used to define a gender-specific starting point in the Third National Health and Nutrition Examination (NHANES III) (9). In the present study, Participants were classified into high- and low-LAP groups at baseline using cut-off points obtained with the baseline receiver operating characteristics analysis, and then subsequently into four transition patterns during follow-up (2011-2015): maintained-high, maintained-low, high-to-low, and low-to-high LAP. The cut-off points of LAP for the diagnosis of T2DM were 18.86 (51.3% specificity and 67.7% sensitivity) for men and 40.53 (70.2% specificity and 51.1% sensitivity) for women (**Figure S3**).

The diagnosis of T2DM was established using criteria from the American Diabetes Association: fasting blood glucose ≥ 126 mg/dL (7.0 mmol/L), and/or random blood glucose ≥ 200 mg/dL (11.1 mmol/L), and/or HbA1c ≥ 6.5%, and/or self-reported diagnosis, and/or currently under hypoglycemic therapy (21).

### Statistical Analysis

Statistical analysis was conducted using SAS statistical software (version 9.4; SAS Institute Inc., Cary, NC, USA). Differences in continuous and categorical variables across groups were assessed by Mann-Whitney test and Chi-square test. Person-years were calculated from the date of baseline interview and physical examination (CHARLS 2011) until the occurrence of T2DM events or death or the time when he/she was censored or the end of follow-up (CHARLS 2018), whichever came first. The cumulative incidence rates of T2DM were calculated by the Kaplan-Meier method. Cox frailty models with random effect and adjustments, were used to explore associations between LAP level and new onset T2DM by gender and setting (rural and urban). Model 1 adjusted for age. Model 2 adjusted for education, marital status, ln (PCE), region, hypertension, smoking, drinking, and general obesity based on Model 1. Model 3 adjusted for TC, LDL-C and HDL-C based on Model 2. In addition, the effects of four transition patterns of LAP on T2DM were assessed by multivariable Cox frailty models. A multiple imputation method was used to impute missing data of baseline characteristics. A total of 945 subjects were imputed for missing data (**Table S3**).

## Results

### Demographic Characteristics

The baseline characteristics of the included participants stratified by gender, setting (rural and urban) and LAP transition pattern are shown in **Table 1** and **Table S4**. In total, 7397 participants (men=3447, women=3950) were involved in cohort analysis. No significant gender differences were observed between rural and urban areas. More women than men were illiterate in both rural and urban settings; the proportion of people with a low education level in rural areas was significantly higher than that in urban settings. The baseline LAP score was 25.46 (interquartile range [IQR]: 14.39-44.41). Totally, 4202 (56.8%) individuals were with low-LAP (men=1734, women=2468) while 3195 (43.2%) were identified as high LAP at baseline (men=1713, women=1482). Significant differences in LAP status were observed between genders in both rural and urban settings. The proportion of general obesity and hypertension was significantly higher in women than in men. Higher plasma HDL and TG were more observed in women compared to men in both rural and urban settings (*P*<0.05).

**Table 1:** Demographic, socioeconomic and geographic characteristics of the included participants at baseline (CHARLS 2011).

Characteristics	Overall (n = 7,397)	Male		*P* ^a^	Female		P^b^
		Rural	Urban		Rural	Urban	
		(n = 2,353)	(n = 1,094)		(n = 2,626)	(n = 1,324)	
**Age group**				0.131			0.156
45-49 years	1437(19.4%)	369(15.7%)	182(16.6%)		566(21.5%)	320(24.2%)	
50-59 years	2641(35.7%)	819(34.8%)	416(38.1%)		942(35.9%)	464(35.0%)	
60-69 years	2182(29.5%)	748(31.8%)	326(29.8%)		759(28.9%)	349(26.4%)	
≥70 years	1137(15.4%)	417(17.7%)	170(15.5%)		359(13.7%)	191(14.4%)	
**Education**				<0.001			<0.001
Illiterate	2173(29.4%)	393(16.7%)	74(6.7%)		1318(50.2%)	388(29.3%)	
Literate	1403(19.0%)	496(21.1%)	203(18.6%)		488(18.6%)	216(16.3%)	
Primary education	1661(22.5%)	699(29.7%)	259(23.7%)		450(17.1%)	253(19.1%)	
Middle school	2159(29.1%)	764(32.5%)	558(51.0%)		370(14.1%)	467(35.3%)	
**Marital status**				<0.001			0.035
Married or cohabiting	6518(88.1%)	2108(89.6%)	1035(94.6%)		2266(86.3%)	1109(83.8%)	
Single	879(11.9%)	245(10.4%)	59(5.4%)		360(13.7%)	215(16.2%)	
**Ln(PCE)**				<0.001			<0.001
Bottom tertile	2164(33.3%)	801(37.9%)	223(23.3%)		887(38.4%)	253(22.6%)	
Middle tertile	2166(33.3%)	704(33.3%)	326(34.0%)		749(32.5%)	387(34.6%)	
Top tertile	2166(33.3%)	607(28.7%)	409(42.7%)		672(29.1%)	478(42.8%)	
**Region of China**				<0.001			0.001
North	950(12.9%)	342(14.5%)	120(11.0%)		328(12.4%)	160(12.0%)	
Northeast	503(6.8%)	136(5.8%)	92(8.4%)		167(6.4%)	108(8.2%)	
East	2221(30.0%)	678(28.8%)	345(31.5%)		793(30.2%)	405(30.6%)	
South Central	1747(23.6%)	486(20.7%)	294(26.9%)		614(23.4%)	353(26.7%)	
Southwest	1366(18.5%)	475(20.2%)	194(17.7%)		474(18.1%)	223(16.8%)	
Northwest	610(8.2%)	236(10.0%)	49(4.5%)		250(9.5%)	75(5.7%)	
**General Obesity*^c^***				<0.001			<0.001
Normal	3657(50.5%)	1452(62.9%)	497(46.2%)		1226(47.8%)	482(37.3%)	
Overweight	2748(37.9%)	715(30.9%)	461(42.8%)		1007(39.2%)	565(43.8%)	
Obese	840(11.6%)	144(6.2%)	118(11.0%)		334(13.0%)	244(18.9%)	
**Hypertension*^c^***				0.009			0.011
Normal	4394(59.5%)	1473(62.8%)	634(58.1%)		1559(59.5%)	728(55.2%)	
Hypertension	2986(40.5%)	874(37.2%)	458(41.9%)		1063(40.5%)	591(44.8%)	
**Smoking**				<0.001			0.030
No Smoking	4506(61.1%)	538(23.0%)	323(29.7%)		2441(93.1%)	1204(91.1%)	
Smoking	2869(38.9%)	1805(77.0%)	766(70.3%)		181(6.9%)	117(8.9%)	
**Drinking*^c^***				0.859			0.007
No Drinking	5089(68.8%)	1046(44.5%)	483(44.1%)		2345(89.3%)	1215(92.0%)	
Drinking	2304(31.2%)	1306(55.5%)	611(55.9%)		281(10.7%)	106(8.0%)	
**TC**				0.291			0.489
≤200mg/dL	4577(61.9%)	1602(68.1%)	725(66.3%)		1506(57.3%)	744(56.2%)	
>200mg/dL	2820(38.1%)	751(31.9%)	369(33.7%)		1120(42.7%)	580(43.8%)	
**HDL**				<0.001			<0.001
≥50mg/dL	3730(50.4%)	1179(50.1%)	437(39.9%)		1460(55.6%)	654(49.4%)	
<50mg/dL	3667(49.6%)	1174(49.9%)	657(60.1%)		1166(44.4%)	670(50.6%)	
**LDL**				0.001			0.253
≤100mg/dL	3214(43.5%)	1199(51.0%)	489(44.7%)		1031(39.3%)	495(37.4%)	
>100mg/dL	4183(56.5%)	1154(49.0%)	605(55.3%)		1595(60.7%)	829(62.6%)	
**TG**				<0.001			0.012
≤150mg/dL	5667(76.6%)	1925(81.8%)	824(75.3%)		1973(75.1%)	945(71.4%)	
>150mg/dL	1730(23.4%)	428(18.2%)	270(24.7%)		653(24.9%)	379(28.6%)	
**LAP SCORE**				<0.001			<0.001
Median (IQR)	25.46(14.39-44.41)	16.90(9.67-30.91)	23.56(12.89-41.87)		30.10(18.47-49.10)	35.59(20.91-57.75)	
**LAP**				<0.001			<0.001
Low LAP	4202(56.8%)	1303(55.4%)	431(39.4%)		1719(65.5%)	749(56.6%)	
High LAP	3195(43.2%)	1050(44.6%)	663(60.6%)		907(34.5%)	575(43.4%)	

Data were presented as n (%) or median with interquartile range (IQR); ^a^comparison between rural males and urban males; ^b^comparison between rural females and urban females; ^c^data for some participants were missing; PCE, per capita expenditures; LAP, lipid accumulation product.

### Association Between Baseline LAP and T2DM

**Figure 1:**
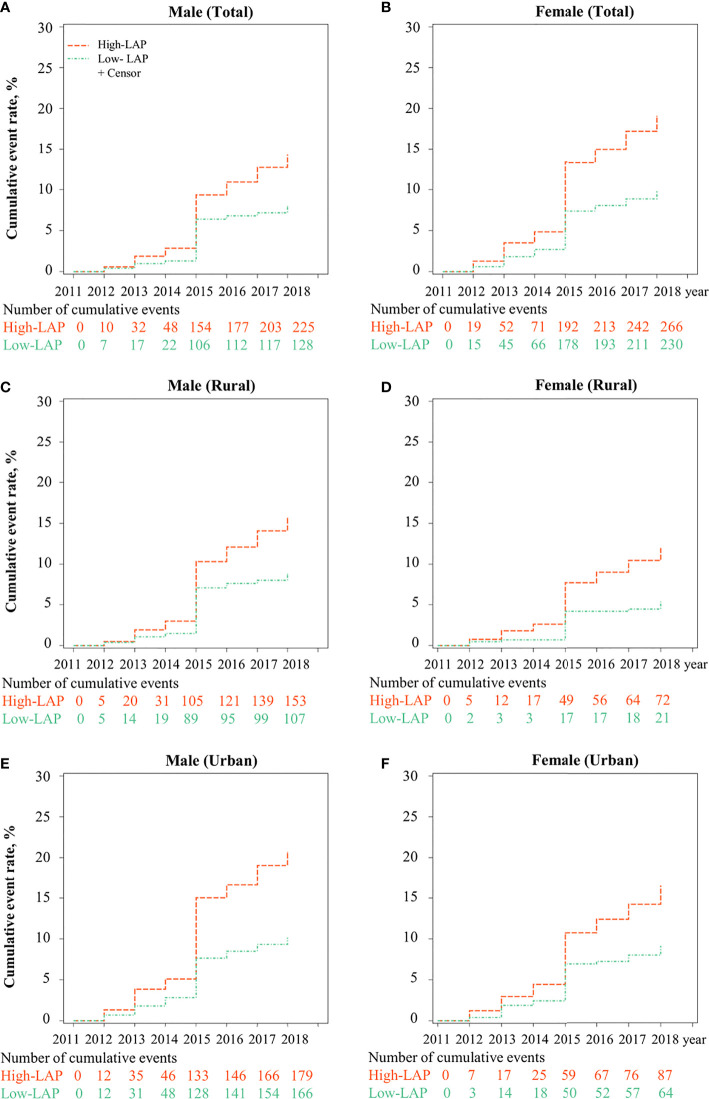
Cumulative incidence of T2DM for LAP phenotypes (Low- and High-) stratified by urban/rural settings and sex from CHARLS 2011 to 2018. *LAP, lipid accumulation product; The cumulative event rate of T2DM was significantly different between low-LAP and high-LAP in* **(A)** *Male*,** (B)** *Female*,** (C)** *Male in rural*,** (D)** *Female in rural*,** (E)** *Male in urban, and* **(F)** *Female in urban*.

**Figure 1** shows the cumulative incidence of T2DM for low- and high-baseline LAP status stratified by setting and gender, from 2011 to 2018. Overall, the cumulative incidence of T2DM was highest in women with high-LAP at baseline (17.95%). Association between baseline LAP status and the risk of new-onset T2DM is shown in **Table 2** and **Table S5**. Positive associations between incident T2DM and high-LAP status at baseline were observed in both genders, with a crude hazard ratios (HR) of 1.78 (95% confidence interval [CI]: 1.43-2.22) for men and =2.00 (95%CI: 1.68-2.39) for women. After full adjustments, the association was still significant in women (HR =1.37, 95%CI: 1.07-1.77) but insignificant in men (HR = 1.29, 95% CI: 0.96-1.74). When further stratified by setting, fully adjusted models revealed significant associations between baseline high-LAP and T2DM only in rural women, with HR being 1.60 (95% CI: 1.18-2.17).

**Table 2:** Hazard ratios for T2DM by LAP phenotypes in middle-aged and older Chinese, CHARLS 2011-2018.

Model	LAP group for Male		LAP group for Female	
	Low LAP	High LAP	Low LAP	High LAP
	(n = 1,734)	(n = 1,713)	(n = 2,468)	(n = 1,482)
**New-onset T2DM**	128 (7.38)	225 (13.13)	230 (9.32)	266 (17.95)
**Overall**				
Unadjusted	1 (reference)	1.78 (1.43, 2.22)^a^	1 (reference)	2.00 (1.68, 2.39)^a^
Model 1	1 (reference)	1.83 (1.47, 2.28)^a^	1 (reference)	1.98 (1.66, 2.36)^a^
Model 2	1 (reference)	1.39 (1.05, 1.85)^a^	1 (reference)	1.51 (1.20, 1.90)^a^
Model 3	1 (reference)	1.29 (0.96, 1.74)	1 (reference)	1.37 (1.07, 1.77)^a^
**Rural**				
Unadjusted	1 (reference)	1.76 (1.37, 2.25)^a^	1 (reference)	2.12 (1.71, 2.62)^a^
Model 1	1 (reference)	1.82 (1.41, 2.33)^a^	1 (reference)	2.11 (1.71, 2.62)^a^
Model 2	1 (reference)	1.29 (0.94, 1.78)	1 (reference)	1.67 (1.27, 2.20)^a^
Model 3	1 (reference)	1.20 (0.86, 1.68)	1 (reference)	1.60 (1.18, 2.17)^a^
**Urban**				
Unadjusted	1 (reference)	2.27 (1.39, 3.69)^a^	1 (reference)	1.84 (1.34, 2.55)^a^
Model 1	1 (reference)	2.30 (1.41, 3.75)^a^	1 (reference)	1.73 (1.25, 2.39)^a^
Model 2	1 (reference)	2.06 (1.11, 3.84)^a^	1 (reference)	1.26 (0.82, 1.93)
Model 3	1 (reference)	1.85 (0.96, 3.59)	1 (reference)	1.02 (0.63, 1.63)

a P < 0.05.Data were presented as n (%) or hazard ratios (95% CI); Associations between LAP and the risk of T2DM were assessed using multivariable Cox frailty models with random intercepts to account for clustering of participants by city; T2DM, type 2 diabetes mellitus; LAP, lipid accumulation product.

Model 1 was adjusted for age. Model 2 was adjusted for education, marital status, Ln(PCE), region, hypertension, smoking, drinking, and general obesity based on Model 1. Model 3 was adjusted for TC, LDL-c and HDL-c based on Model 2.

### Association Between LAP Transition and T2DM

**Figure 2:**
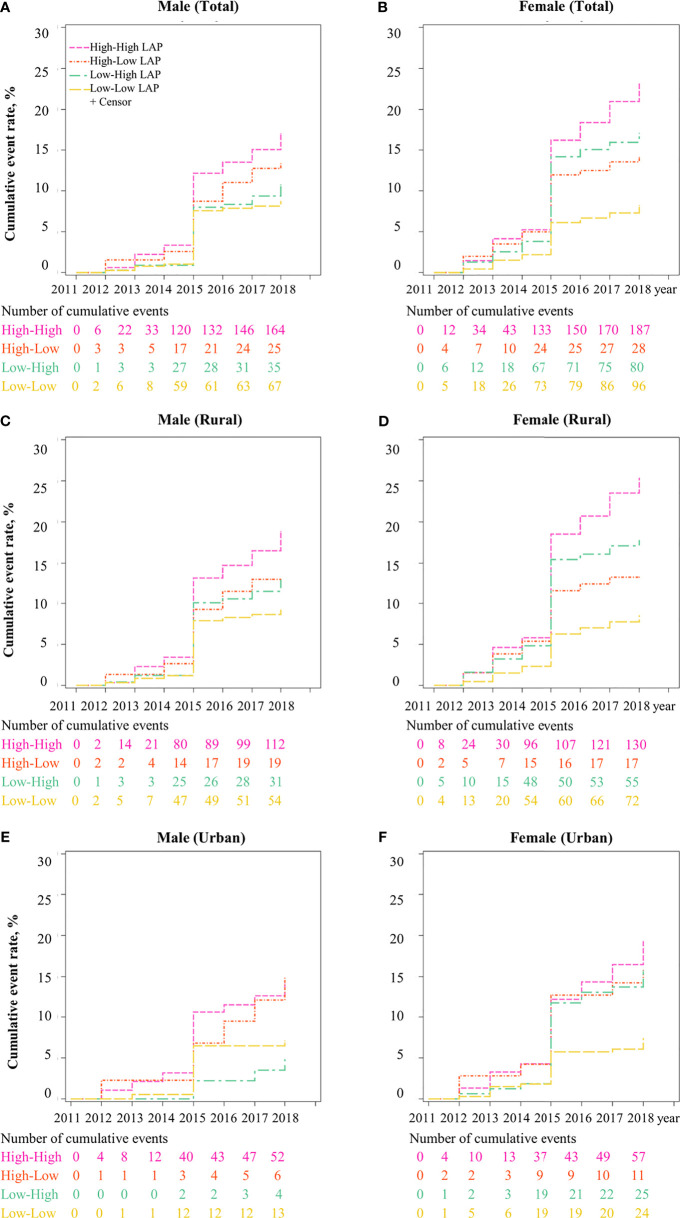
Cumulative incidence of T2DM for LAP transitions from CHARLS 2011 to 2018. Note: LAP, lipid accumulation product; The cumulative event rate of T2DM was significantly different across four LAP transitions (high to high-, high to low-, low to high-, low to low-) in **(A)** Male, **(B)** Female, **(C)** Male in rural, **(D)** Female in rural, **(E)** Male in urban, and **(F)** Female in urban.

**Figure 3:**
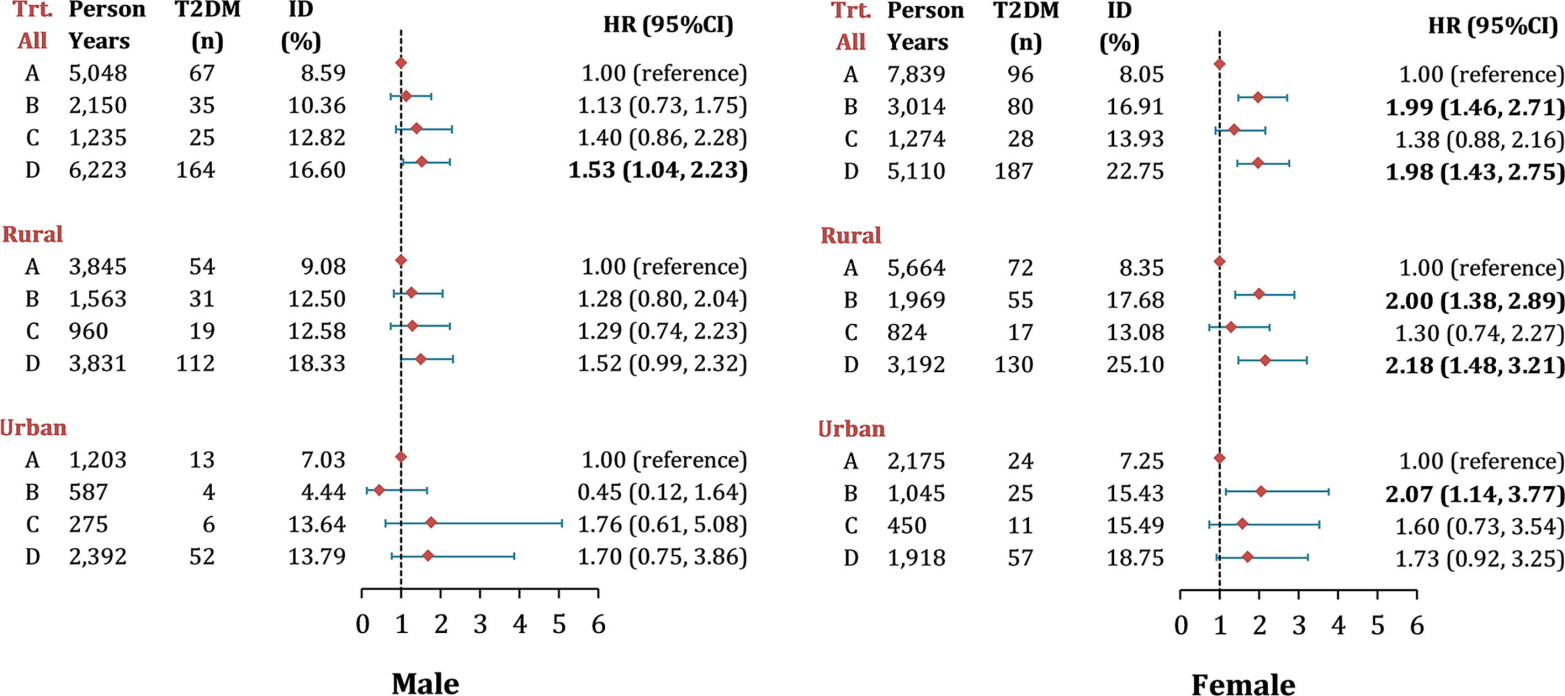
Risk of new-onset T2DM by different LAP transitions in middle-aged and older Chinese. Note: Data were presented as n (%) and hazard ratios (95% CI), adjusted for age, education, region, hypertension, smoking, drinking, general obesity, TC, LDL-c level and HDL-c level; Hazard ratios for T2DM by LAP transitions were calculated using multivariable Cox frailty models with random effect, by which means clustering of participants was accounted for; P< 0.05 were highlighted in bold. ID, incidence density; LAP, lipid accumulation product; Trt., transition types during follow-up, the definition from group A to D were listed as following: Group A, maintain Low LAP during follow-up; Group B, Low LAP at baseline turned to High LAP at follow-up; Group C, High LAP at baseline turned to Low LAP at follow-up; Group D, maintain High LAP during follow-up.

In terms of LAP transitions, it was found that the cumulative incidence of T2DM was the highest in participants with maintained-high (**Figure 2**). **Figure 3** reports the risk of T2DM by LAP transition pattern stratified by gender after adjusting for age, education, region, hypertension, smoking, drinking, general obesity, TC, LDL-C and HDL-C. Overall, compared to individual whose LAP level was maintained low from baseline to follow-up, a 1.5 to 2.0-fold risk of T2DM was observed in people with maintained-high LAP pattern (HR_men_ = 1.53, 95 CI%: 1.04-2.23, HR_women_ = 1.98, 95% CI: 1.43-2.75, *P*<0.05), and a two-fold risk of T2DM was observed in women whose LAP level was transferred from low to high (HR = 1.99, 95% CI: 1.46-2.71). Comparatively, male participants with three transition patterns (low-to-high, high-to-low, and maintained-high) showed no significant difference in the risk of T2DM in both urban and rural settings, compared to those who have maintained-low LAP pattern during the follow-up. Similar results were found in **Tables S6, S7** for data with multiple imputations.

## Discussion

In this prospective cohort study, we confirmed that higher LAP level was a significant risk factor for T2DM, especially in women. Women with high-LAP status at baseline presented a higher risk of T2DM. When assessing the relationship between LAP transitions and T2DM, women with maintained-high LAP pattern and low-to-high transition pattern were at an almost two-fold risk of T2DM compared to maintained-low LAP group. However, no such association was generally found in men, except for the maintained-high LAP pattern.

A number of studies have reported the rapid increase in the prevalence of DM in the Chinese adult population (22–24). The disease burden of DM in China is likely the result of population aging, urbanization, unhealthy diets, reduced exercise, and the consequent epidemic of obesity (25). Although visceral fat has been found to be independently associated with insulin resistance and could be used to estimate the risk of T2DM, the measurement for body visceral adiposity tissue volumes is not easy to conduct. MRI and CT have been considered the gold standard for visceral adiposity measurements, but both are not suitable for large epidemiological studies due to high costs and inconvenience. Comparatively, LAP can be easily measured in large-scale epidemiological studies and has been suggested as a useful surrogate marker of visceral adiposity. Previous studies have demonstrated that LAP has a similar or greater capacity to predict T2DM when compared with common fatness indices, such as BMI, WC, WHR and WHtR (10–12). For example, a six-year cohort study showed that LAP performed as good as BMI, WHR, and WHtR when predicting T2DM (26). Findings from this study were consistent with previous results, which indicated the positive effects of LAP on the prediction of DM in both genders (9, 26–28). Furthermore, in a recent cohort study examining the potential of LAP as an indicator for the development of T2DM among 15717 Chinese individuals (28), results showed a significantly increased risk of T2DM development in high LAP groups.

As for the effects of LAP by gender and by rural/urban setting, this study observed that the cumulative incidence of T2DM in women with high-LAP status was the highest. Similar results were found in a large population-based study in Japan (29), which reported the odds ratio for DM in subjects with high LAP was higher in women (OR=19.09) than in men (OR=7.40). A recent meta-analysis demonstrated a higher prevalence of T2DM in rural Chinese women than that in men (30). The difference in T2DM prevalence between genders was presumably explained by two reasons. The first might be the difference in sex steroid hormones and glucose homeostasis (31). Since the fat mass in healthy women is higher than that in men, the circulating free fatty acids and intramuscular fat content would consequently be higher (32), which could therefore induce insulin resistance in women. Another explanation might be the lack of physical activity in women compared to men. A regional study in China showed that rural men presented a significantly lower risk of developing metabolic syndrome than rural women and the low risks were observed in those who were less sitting and engaged in more vigorous physical activity (33). Moreover, randomized clinical trials have shown that interventions involving exercise were significantly associated with a reduced risk of DM among people with prediabetes (34–36). Therefore, it is important that future research investigates the lifestyles of women in rural China, public health measures are also needed to mitigate the consequences of new cases of T2DM in this group.

Interestingly, for female participants, when assessing the impact of LAP transition on T2DM, the low-to-high LAP group had a similar risk for developing T2DM compared to the maintained-high LAP group, both groups were significantly higher than the maintained-low LAP group; comparatively, no significant difference was found between high-to-low LAP group and maintained-low LAP group. These findings may partly be explained by the “vicious circle” - the dyslipidemia-insulin resistance-hyperinsulinemia circle - in the development of T2DM (30). In this vicious circle, the consistently high level of TG, which leads to a maintained-high LAP, contributes to T2DM by competing with glucose to enter the cell, decreasing the activity of insulin receptors on fat cells, and preventing insulin from combining with receptors; moreover, the high LAP could also attribute to the decreased level of HDL, which can negatively influence the β cell’s function in the pancreas and reduce insulin sensitivity (31). Conversely, insulin resistance could result in the increasing of TG and decreasing of HDL. Given those, an increase in LAP from a low to a high level may be associated with the development of T2DM. Meanwhile, reducing and maintaining LAP at a lower level would be vital for the prevention of T2DM. Hence, further intervention studies could consider ways of shifting LAP levels as a way of preventing T2DM, especially for women.

This study is the first to analyze the impact of LAP and its transition on incident T2DM in middle to older aged population. Compared to previous studies, the sample of this study was relatively large, and representative based on participants from multiple regions in China. One of the limitations in the present study is that, due to the original design of the survey, blood sample data were only available in 2011 and 2015, which brought sudden increases in incident T2DM in 2015. Moreover, several confounding factors, such as family history and diet were not accounted for in our multivariable analyses due to the absence of relative information in the CHARLS dataset. Additionally, considering over half of the participants were excluded from this study, selection bias may be introduced.

This study demonstrated that high LAP was associated with the development of T2DM among Chinese women over 45 years old. The maintained high LAP and transition of LAP from low to high levels were confirmed as risk factors for T2DM. Future efforts aimed at preventing T2DM should be made to explore ways of decreasing LAP levels.

## Data Availability Statement

Publicly available datasets were analyzed in this study. This data can be found here: http://charls.pku.edu.cn/pages/data/111/en.html.

## Ethics Statement

The studies involving human participants were reviewed and approved by Ethics Review Committee of Peking University. The patients/participants provided their written informed consent to participate in this study.

## Author Contributions

Conception or design: PS. Acquisition, analysis, or interpretation of data: GC, QY, and JY. Drafting the work or revising: JY, QY, YaS, YuS, LH, and YZ. Final approval of the manuscript: PS, YaS, YuS, LH, YZ, JY, and QY. All authors contributed to the article and approved the submitted version.

## Conflict of Interest

The authors declare that the research was conducted in the absence of any commercial or financial relationships that could be construed as a potential conflict of interest.

## Publisher’s Note

All claims expressed in this article are solely those of the authors and do not necessarily represent those of their affiliated organizations, or those of the publisher, the editors and the reviewers. Any product that may be evaluated in this article, or claim that may be made by its manufacturer, is not guaranteed or endorsed by the publisher.
