# Supplementary material for: Transition of Lipid Accumulation Product Status and the Risk of Type 2 Diabetes Mellitus in Middle-Aged and Older Chinese: A National Cohort Study
Source: Front Endocrinol (Lausanne). 2021 Nov 26;12:770200. doi: 10.3389/fendo.2021.770200 (PMC8660859; doi:10.3389/fendo.2021.770200)
Supplement: Supplementary file 1 [file DataSheet_1.docx]

**Transition of Lipid Accumulation Product Status and the Risk of Type 2 Diabetes Mellitus in Middle-Aged and Older Chinese: a National Cohort Study**

**
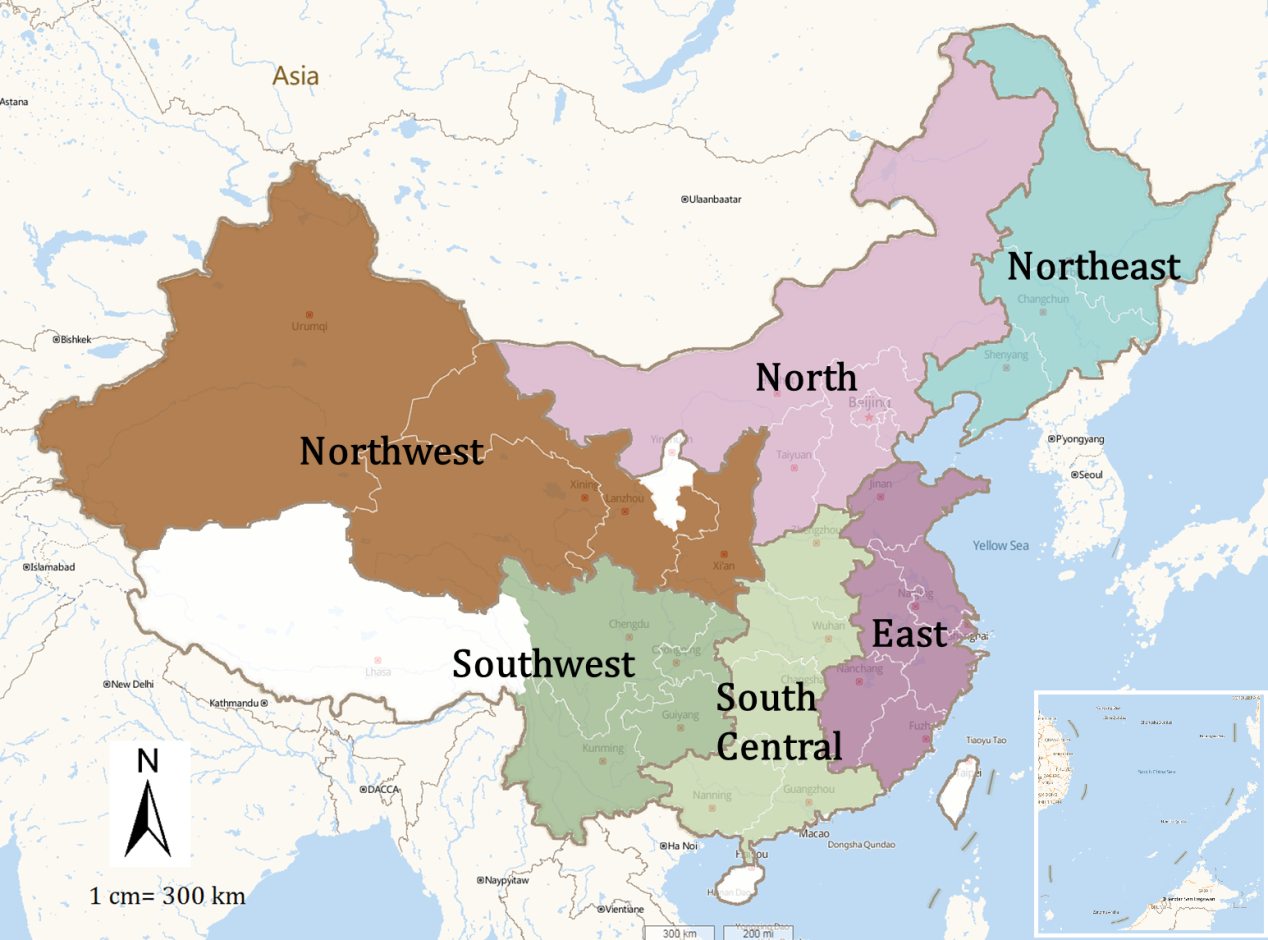
Supplementary Figure S1.** The six geographic regions in China

*Note: Twenty-eight provinces in Mainland China were randomly chosen in CHARLS, except for Hainan province, Ningxia Hui Autonomous Region and Tibet province, which were marked in white.*

**Supplementary Table S1.** Investigated provinces in six geographic regions in CHARLS

| **Region** | **Included provinces** |
| --- | --- |
| North | Beijing Municipality, Hebei province, Inner Mongolia Autonomous Region, Shanxi province, Tianjin Municipality; |
| Northeast | Heilongjiang province, Jilin province, Liaoning province; |
| East | Anhui province, Fujian province, Jiangsu province, Jiangxi province, Shandong province, Shanghai Municipality, Zhejiang province; |
| South Central | Guangdong province, Guangxi Zhuang Autonomous Region, Hainan province, Henan province, Hubei province, Hunan province; |
| Southwest | Chongqing Municipality, Guizhou province, Sichuan province, Tibet Autonomous Region, Yunnan province; |
| Northwest | Gansu province, Ningxia Hui Autonomous Region, Qinghai province, Shaanxi province, Xinjiang Uyghur Autonomous Region; |

*Note: Twenty-eight provinces in Mainland China were randomly chosen in CHARLS, except for Hainan province, Ningxia Hui Autonomous Region and Tibet province.*


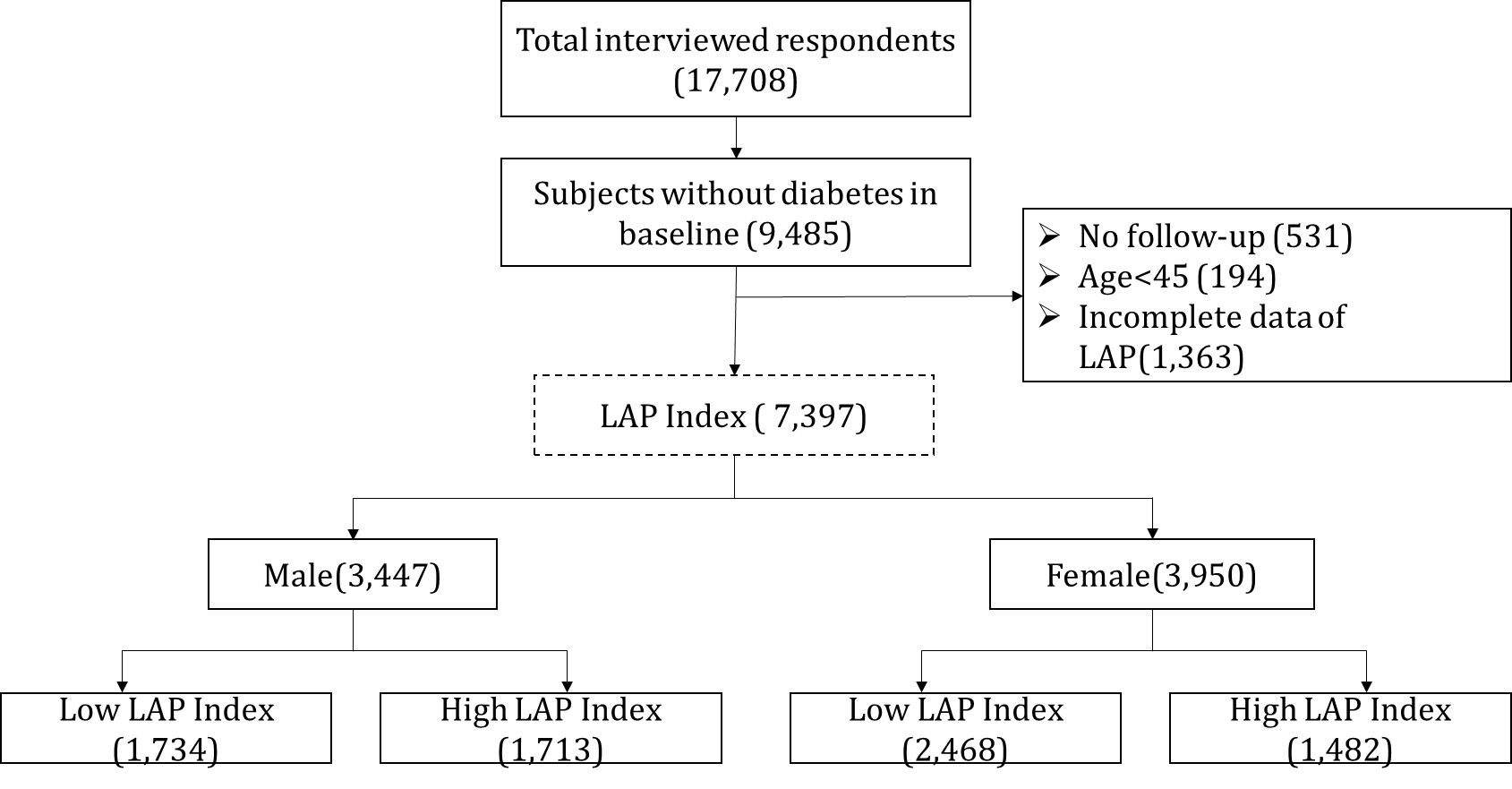


**Supplementary Figure S2.** Flow chart for subjects included in this study

*Note: Incomplete data referred to any incompleteness information of age, gender, educational attainment, marital status, setting, economic status, geographic region; LAP, lipid accumulation product.*

**Supplementary Table S2.** Comparison of general characteristics between the included and non-included subjects in the CHARLS 2011 survey

| **Characteristic** | | **Sample** | **Excluded subjects** | **Included subjects** | ***P**** |
| --- | --- | --- | --- | --- | --- |
|  |  | **(n=17,311)** | **(n=9,914)** | **(n=7,397)** |  |
| **Age group** | |  |  |  | <0.001 |
|  | 45-49 years | 3496 (20.2%) | 2059 (20.8%) | 1437 (19.4%) |  |
|  | 50-59 years | 6128 (35.4%) | 3487 (35.2%) | 2641 (35.7%) |  |
|  | 60-69 years | 4756 (27.5%) | 2574 (26.0%) | 2182 (29.5%) |  |
|  | ≥70 years | 2931 (16.9%) | 1794 (18.0%) | 1137 (15.4%) |  |
| **Gender** | |  |  |  | <0.001 |
|  | Male | 8471 (47.9%) | 5024 (48.8%) | 3447 (46.6%) |  |
|  | Female | 9221 (52.1%) | 5271 (51.2%) | 3950 (53.4%) |  |
| **Rural/Urban** | |  |  |  | <0.001 |
|  | Rural | 10305 (59.5%) | 5558 (53.9%) | 4979 (67.3%) |  |
|  | Urban | 7006 (40.5%) | 4750 (46.1%) | 2418 (32.7%) |  |
| **Education** | |  |  |  | <0.001 |
|  | Illiterate | 4803 (27.2%) | 2630 (25.6%) | 2173 (29.4%) |  |
|  | Literate | 3140 (17.8%) | 1737 (16.9%) | 1403 (19.0%) |  |
|  | Primary education | 3812 (21.6%) | 2151 (21.0%) | 1661 (22.5%) |  |
|  | Middle school | 5898 (33.4%) | 3739 (36.5%) | 2159 (29.1%) |  |
| **Marital status** | |  |  |  | 0.001 |
|  | Married or cohabiting | 15451 (87.4%) | 8933 (86.9%) | 6518 (88.1%) |  |
|  | Single | 2221 (12.6%) | 1342 (13.1%) | 879 (11.9%) |  |
| **Ln(PCE) by setting** | |  |  |  | <0.001 |
|  | Bottom tertile | 4473 (29.8%) | 2309 (27.1%) | 2164 (33.3%) |  |
|  | Middle tertile | 4749 (31.7%) | 2583 (30.4%) | 2166 (33.3%) |  |
|  | Top tertile | 5782 (38.5%) | 3616 (42.5%) | 2166 (33.3%) |  |
| **Region** | |  |  |  | <0.001 |
|  | North China | 2399 (13.5%) | 1449 (14.1%) | 950 (12.9%) |  |
|  | Northeast China | 1306 (7.4%) | 803 (7.8%) | 503 (6.8%) |  |
|  | East China | 5352 (30.2%) | 3131 (30.4%) | 2221 (30.0%) |  |
|  | South Central China | 4351 (24.6%) | 2604 (25.3%) | 1747 (23.6%) |  |
|  | Southwest China | 3023 (17.1%) | 1657 (16.1%) | 1366 (18.5%) |  |
|  | Northwest China | 1274 (7.2%) | 664 (6.4%) | 610 (8.2%) |  |
| **Central Obesity** | |  |  |  | <0.001 |
|  | Normal | 8112 (59.9%) | 3501 (57.0%) | 4611 (62.3%) |  |
|  | Central Obesity | 5424 (40.1%) | 2638 (43.0%) | 2786 (37.7%) |  |
| **General Obesity** | |  |  |  | <0.001 |
|  | Normal | 6565 (48.9%) | 2908 (47.0%) | 3657 (50.5%) |  |
|  | Overweight | 5144 (38.3%) | 2396 (38.7%) | 2748 (37.9%) |  |
|  | Obesity | 1725 (12.8%) | 885 (14.3%) | 840 (11.6%) |  |
| **Hypertension** | |  |  |  | 0.038 |
|  | Normal | 10649 (60.9%) | 6255 (61.8%) | 4394 (59.5%) |  |
|  | Hypertension | 6850 (39.1%) | 3864 (38.2%) | 2986 (40.5%) |  |
| **Smoking** | |  |  |  | 0.006 |
|  | No Smoking | 10626 (62.9%) | 6120 (64.2%) | 4506 (61.1%) |  |
|  | Smoking | 6279 (37.1%) | 3410 (35.8%) | 2869 (38.9%) |  |
| **Drinking** | |  |  |  | 0.577 |
|  | No Drinking | 12113 (69.1%) | 7024 (69.2%) | 5089 (68.8%) |  |
|  | Drinking | 5426 (30.9%) | 3122 (30.8%) | 2304 (31.2%) |  |
| **TC** | |  |  |  | <0.001 |
|  | ≤200mg/dL | 7091 (60.8%) | 2514 (59.0%) | 4577 (61.9%) |  |
|  | >200mg/dL | 4564 (39.2%) | 1744 (41.0%) | 2820 (38.1%) |  |
| **HDL** | |  |  |  | <0.001 |
|  | ≥50mg/dL | 5493 (47.1%) | 1763 (41.3%) | 3730 (50.4%) |  |
|  | <50mg/dL | 6170 (52.9%) | 2503 (58.7%) | 3667 (49.6%) |  |
| **LDL** | |  |  |  | 0.125 |
|  | ≤100mg/dL | 3845 (33.0%) | 1458 (34.3%) | 2387 (32.3%) |  |
|  | >100mg/dL | 7797 (67.0%) | 2789 (65.7%) | 5008 (67.7%) |  |
| **TG** | |  |  |  | <0.001 |
|  | ≤150mg/dL | 8486 (72.8%) | 2819 (66.2%) | 5667 (76.6%) |  |
|  | >150mg/dL | 3170 (27.2%) | 1440 (33.8%) | 1730 (23.4%) |  |

*Note: Data were presented as n (%); P****^*^,*** *comparison between included and excluded subjects; PCE, per capita expenditures; TC, total cholesterol; HDL-C, high-density lipoprotein cholesterol; LDL-C, low-density lipoprotein cholesterol; TG, triglyceride.*

**
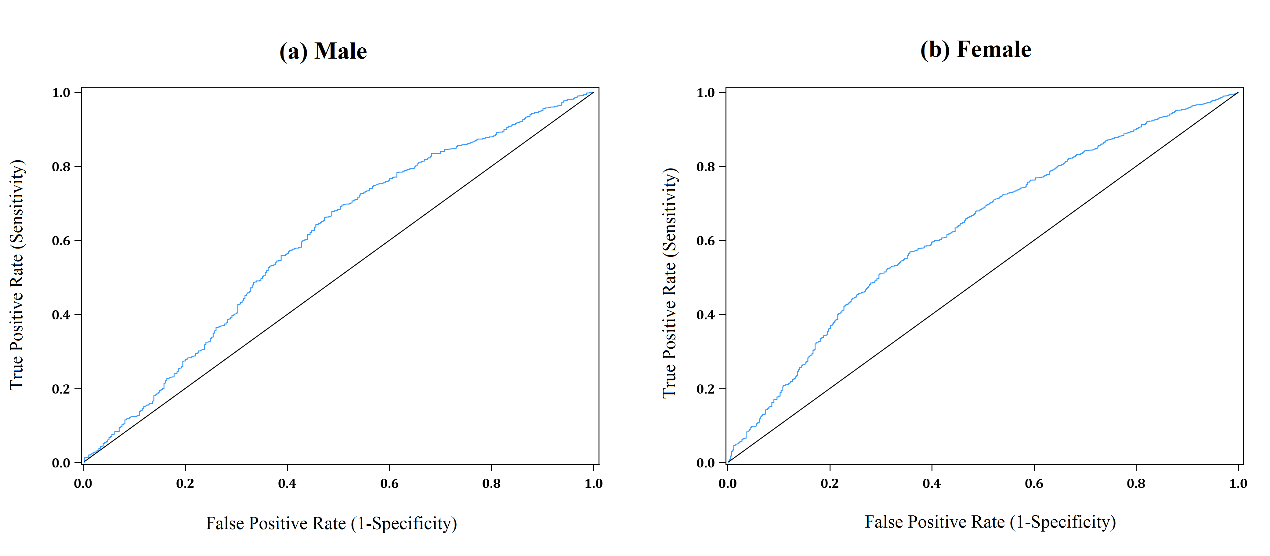
**

**Supplementary Figure S3.** Sex-specific ROC curves of LAP

**Supplementary Table S3.** Number of missing values and corresponding dispositions

| **Characteristic** | **No. of missing values** | **Percent(%)** | **Disposition** |
| --- | --- | --- | --- |
| Education | 1 | 0.01 | Multiple Imputation |
| Ln(PCE) by setting | 901 | 12.18 | Multiple Imputation |
| Hypertension | 17 | 0.23 | Multiple Imputation |
| Smoking | 22 | 0.30 | Multiple Imputation |
| Alcohol drinking | 4 | 0.05 | Multiple Imputation |
| Total | 945 | 12.78 | Multiple Imputation |

**Table S4.** Baseline demographic, socioeconomic and geographic characteristics of the included participants by transition pattern (CHARLS 2011)

| **Characteristic** | | **LAP transition patterns** | | | | ***P^†^*** |
| --- | --- | --- | --- | --- | --- | --- |
|  |  | **Low-Low** | **Low-High** | **High-Low** | **High-High** |  |
|  |  | **(n=1973)** | **(n=811)** | **(n=396)** | **(n=1810)** |  |
| **Age group** | |  |  |  |  | 0.010 |
|  | 45-49 years | 379 (19.2%) | 167 (20.6%) | 60 (15.2%) | 349 (19.3%) |  |
|  | 50-59 years | 679 (34.4%) | 309 (38.1%) | 150 (37.9%) | 685 (37.8%) |  |
|  | 60-69 years | 623 (31.6%) | 229 (28.2%) | 135 (34.1%) | 574 (31.7%) |  |
|  | ≥70 years | 292 (14.8%) | 106 (13.1%) | 51 (12.9%) | 202 (11.2%) |  |
| **Sex** | |  |  |  |  | <0.001 |
|  | Male | 780 (39.5%) | 338 (41.7%) | 195 (49.2%) | 988 (54.6%) | |
|  | Female | 1193 (60.5%) | 473 (58.3%) | 201 (50.8%) | 822 (45.4%) | |
| **Education** | |  |  |  |  | <0.001 |
|  | Illiterate | 656 (33.2%) | 250 (30.9%) | 114 (28.8%) | 414 (22.9%) | |
|  | Literate | 410 (20.8%) | 142 (17.5%) | 79 (19.9%) | 314 (17.3%) | |
|  | Primary education | 414 (21.0%) | 190 (23.5%) | 95 (24.0%) | 464 (25.6%) | |
|  | Middle or higher education | 493 (25.0%) | 228 (28.1%) | 108 (27.3%) | 618 (34.1%) | |
| **Marital status** | |  |  |  |  | <0.001 |
|  | Married or cohabiting | 1722 (87.3%) | 731 (90.1%) | 347 (87.6%) | 1656 (91.5%) | |
|  | Single | 251 (12.7%) | 80 (9.9%) | 49 (12.4%) | 154 (8.5%) | |
| **Ln(PCE)** | | |  |  |  | 0.007 |
|  | Bottom tertile | 631 (36.6%) | 259 (36.5%) | 126 (35.5%) | 509 (31.5%) | |
|  | Middle tertile | 599 (34.7%) | 227 (32.0%) | 115 (32.4%) | 546 (33.8%) | |
|  | Top tertile | 496 (28.7%) | 223 (31.5%) | 114 (32.1%) | 559 (34.6%) | |
| **Region** | |  |  |  |  | <0.001 |
|  | North China | 173 (8.8%) | 100 (12.3%) | 60 (15.2%) | 311 (17.2%) | |
|  | Northeast China | 100 (5.1%) | 69 (8.5%) | 38 (9.6%) | 132 (7.3%) | |
|  | East China | 663 (33.6%) | 249 (30.7%) | 134 (33.8%) | 570 (31.5%) | |
|  | South Central China | 489 (24.8%) | 175 (21.6%) | 75 (18.9%) | 416 (23.0%) | |
|  | Southwest China | 357 (18.1%) | 146 (18.0%) | 55 (13.9%) | 234 (12.9%) | |
|  | Northwest China | 191 (9.7%) | 72 (8.9%) | 34 (8.6%) | 147 (8.1%) | |
| **General Obesity** | |  |  |  |  | <0.001 |
|  | Normal | 1489 (76.3%) | 395 (49.7%) | 179 (45.4%) | 349 (19.9%) | |
|  | Overweight | 430 (22.0%) | 355 (44.7%) | 160 (40.6%) | 932 (53.1%) | |
|  | Obesity | 33 (1.7%) | 44 (5.5%) | 55 (14.0%) | 474 (27.0%) | |
| **Central Obesity** | |  |  |  |  | <0.001 |
|  | Normal | 1684 (85.4%) | 578 (71.3%) | 217 (54.8%) | 575 (31.8%) | |
|  | Central obesity | 289 (14.6%) | 233 (28.7%) | 179 (45.2%) | 1235 (68.2%) | |
| **Hypertension** | |  |  |  |  | <0.001 |
|  | Normal | 1398 (71.1%) | 516 (63.7%) | 222 (56.2%) | 855 (47.3%) | |
|  | Hypertension | 569 (28.9%) | 294 (36.3%) | 173 (43.8%) | 951 (52.7%) | |
| **Smoking** | |  |  |  |  | <0.001 |
|  | Non-smoker | 1286 (65.2%) | 517 (64.1%) | 239 (60.5%) | 1027 (57.0%) | |
|  | Smoker | 686 (34.8%) | 289 (35.9%) | 156 (39.5%) | 776 (43.0%) | |
| **Alcohol drinking** | |  |  |  |  | <0.001 |
|  | Non-drinker | 1426 (72.3%) | 586 (72.3%) | 271 (68.4%) | 1174 (64.9%) | |
|  | Drinker | 545 (27.7%) | 225 (27.7%) | 125 (31.6%) | 635 (35.1%) | |
| **TC** | |  |  |  |  | <0.001 |
|  | ≤200mg/dL | 1374 (69.6%) | 510 (62.9%) | 228 (57.6%) | 994 (54.9%) | |
|  | >200mg/dL | 599 (30.4%) | 301 (37.1%) | 168 (42.4%) | 816 (45.1%) | |
| **HDL** | |  |  |  |  | <0.001 |
|  | ≥50mg/dL | 1414 (71.7%) | 462 (57.0%) | 146 (36.9%) | 458 (25.3%) | |
|  | <50mg/dL | 559 (28.3%) | 349 (43.0%) | 250 (63.1%) | 1352 (74.7%) | |
| **LDL** | |  |  |  |  | <0.001 |
|  | ≤100mg/dL | 1042 (52.8%) | 344 (42.4%) | 182 (46.0%) | 839 (46.4%) | |
|  | >100mg/dL | 931 (47.2%) | 467 (57.6%) | 214 (54.0%) | 971 (53.6%) | |
| **TG** | |  |  |  |  | <0.001 |
|  | ≤150mg/dL | 1916 (97.1%) | 769 (94.8%) | 225 (56.8%) | 893 (49.3%) | |
|  | >150mg/dL | 57 (2.9%) | 42 (5.2%) | 171 (43.2%) | 917 (50.7%) |  |

*Note: Data were presented as n (%); P****^†^,*** *comparison between four LAP transition patterns; LAP, lipid accumulation product; PCE, per capita expenditures; TC, total cholesterol; HDL-C, high-density lipoprotein cholesterol; LDL-C, low-density lipoprotein cholesterol; TG, triglyceride.*

**Supplementary Table S5.** Multivariable Cox frailty models on associated factors and T2DM

| **Characteristic** | | **T2DM for Male** | | |  | **T2DM for Female** | | |
| --- | --- | --- | --- | --- | --- | --- | --- | --- |
|  |  | **Model 1** | **Model 2** | **Model 3** |  | **Model 1** | **Model 2** | **Model 3** |
| **Age group** | |  |  |  |  |  |  |  |
|  | 45-49 years | 1 (reference) | 1 (reference) | 1 (reference) |  | 1 (reference) | 1 (reference) | 1 (reference) |
|  | 50-59 years | 1.75(1.20, 2.54)* | 1.67(1.12, 2.49)* | 1.68(1.12, 2.50)* |  | 1.27(0.97, 1.64) | 1.02(0.76, 1.38) | 0.99(0.73, 1.34) |
|  | 60-69 years | 2.11(1.45, 3.07)* | 1.91(1.26, 2.89)* | 1.93(1.28, 2.92)* |  | 1.54(1.19, 2.01)* | 1.22(0.89, 1.67) | 1.19(0.86, 1.63) |
|  | ≥70 years | 2.00(1.31, 3.06)* | 1.86(1.15, 3.02)* | 1.94(1.20, 3.15)* |  | 1.47(1.07, 2.02)* | 1.20(0.81, 1.80) | 1.17(0.78, 1.75) |
| **Education** | |  |  |  |  |  |  |  |
|  | Illiterate | NA | 1 (reference) | 1 (reference) |  | NA | 1 (reference) | 1 (reference) |
|  | Literate | NA | 1.03(0.68, 1.58) | 1.03(0.67, 1.58) |  | NA | 1.01(0.77, 1.32) | 1.01(0.77, 1.32) |
|  | Primary education | NA | 1.12(0.75, 1.68) | 1.12(0.75, 1.68) |  | NA | 0.64(0.47, 0.87)* | 0.64(0.47, 0.88)* |
|  | Middle school | NA | 0.98(0.65, 1.47) | 0.99(0.65, 1.49) |  | NA | 0.71(0.52, 0.97)* | 0.70(0.51, 0.96)* |
| **Marital status** | |  |  |  |  |  |  |  |
|  | Married or cohabiting | NA | 1 (reference) | 1 (reference) |  | NA | 1 (reference) | 1 (reference) |
|  | Single | NA | 1.00(0.66, 1.53) | 1.00(0.65, 1.52) |  | NA | 1.18(0.88, 1.59) | 1.20(0.89, 1.62) |
| **Ln(PCE) by setting** | | |  |  |  |  |  |  |
|  | Bottom tertile | NA | 1 (reference) | 1 (reference) |  | NA | 1 (reference) | 1 (reference) |
|  | Middle tertile | NA | 1.04(0.79, 1.37) | 1.04(0.78, 1.37) |  | NA | 0.89(0.70, 1.13) | 0.89(0.70, 1.14) |
|  | Top tertile | NA | 0.99(0.74, 1.33) | 1.00(0.75, 1.35) |  | NA | 0.95(0.74, 1.22) | 0.95(0.74, 1.22) |
| **Region** | |  |  |  |  |  |  |  |
|  | North China | NA | 1 (reference) | 1 (reference) |  | NA | 1 (reference) | 1 (reference) |
|  | Northeast China | NA | 0.54(0.29, 1.01) | 0.54(0.29, 1.01) |  | NA | 0.50(0.30, 0.85)* | 0.50(0.30, 0.84)* |
|  | East China | NA | 0.78(0.53, 1.16) | 0.79(0.53, 1.18) |  | NA | 0.76(0.55, 1.05) | 0.75(0.54, 1.05) |
|  | Southcentral China | NA | 0.77(0.51, 1.16) | 0.77(0.50, 1.17) |  | NA | 0.78(0.55, 1.10) | 0.77(0.54, 1.09) |
|  | Southwest China | NA | 0.71(0.45, 1.12) | 0.73(0.46, 1.15) |  | NA | 0.75(0.52, 1.09) | 0.75(0.51, 1.10) |
|  | Northwest China | NA | 1.11(0.67, 1.84) | 1.11(0.66, 1.86) |  | NA | 0.77(0.49, 1.22) | 0.78(0.49, 1.24) |
| **Hypertension** | |  |  |  |  |  |  |  |
|  | Never | NA | 1 (reference) | 1 (reference) |  | NA | 1 (reference) | 1 (reference) |
|  | Hypertension | NA | 1.32(1.04, 1.68)* | 1.32(1.04, 1.68)* |  | NA | 1.28(1.04, 1.58)* | 1.29(1.04, 1.59)* |
| **Obesity** | |  |  |  |  |  |  |  |
|  | Normal | NA | 1 (reference) | 1 (reference) |  | NA | 1 (reference) | 1 (reference) |
|  | Overweight | NA | 1.16(0.87, 1.54) | 1.15(0.87, 1.53) |  | NA | 1.45(1.13, 1.85)* | 1.44(1.12, 1.84)* |
|  | Obesity | NA | 1.59(1.06, 2.39)* | 1.53(1.02, 2.31)* |  | NA | 1.77(1.29, 2.44)* | 1.76(1.28, 2.42)* |
| **Smoking** | |  |  |  |  |  |  |  |
|  | No Smoking | NA | 1 (reference) | 1 (reference) |  | NA | 1 (reference) | 1 (reference) |
|  | Smoking | NA | 1.12(0.85, 1.47) | 1.11(0.84, 1.46) |  | NA | 1.17(0.80, 1.71) | 1.16(0.79, 1.69) |
| **Drinking** | |  |  |  |  |  |  |  |
|  | No drinking | NA | 1 (reference) | 1 (reference) |  | NA | 1 (reference) | 1 (reference) |
|  | drinking | NA | 1.06(0.83, 1.34) | 1.07(0.84, 1.36) |  | NA | 0.93(0.65, 1.32) | 0.93(0.66, 1.33) |
| **TC** | |  |  |  |  |  |  |  |
|  | ≤200mg/dL | NA | NA | 1 (reference) |  | NA | NA | 1 (reference) |
|  | >200mg/dL | NA | NA | 1.33(1.02, 1.73)* |  | NA | NA | 1.11(0.89, 1.39) |
| **LDL** | |  |  |  |  |  |  |  |
|  | ≤100mg/dL | NA | NA | 1 (reference) |  | NA | NA | 1 (reference) |
|  | >100mg/dL | NA | NA | 0.80(0.62, 1.02) |  | NA | NA | 1.09(0.87, 1.36) |
| **HDL** | |  |  |  |  |  |  |  |
|  | ≥50mg/dL | NA | NA | 1 (reference) |  | NA | NA | 1 (reference) |
|  | <50mg/dL | NA | NA | 1.22(0.94, 1.59) |  | NA | NA | 1.20(0.96, 1.51) |

*Note: Data were presented as hazard ratios (95% CI); Associations between LAP and T2DM were conducted using multivariable Cox frailty models with random effect to account for clustering of participants in each province; T2DM, type 2 diabetes mellitus; LAP, lipid accumulation product; PCE, per capita expenditures; TC, total cholesterol; HDL-C, high-density lipoprotein cholesterol; LDL-C, low-density lipoprotein cholesterol; TG, triglyceride;* *NA, not available; * , P<0.05.*

*Model 1 was adjusted for age. Model 2 was adjusted for education, marital status, Ln(PCE) by setting, region, hypertension, smoking, drinking, and general obesity based on Model 1. Model 3 was adjusted for TC, LDL-C and HDL-C based on Model 2.*

**Supplementary Table S6.** Hazard ratios for T2DM by LAP status in middle-aged and older Chinese based on multiply imputed datasets

| **Characteristic** | | **T2DM for Male** | | |  | **T2DM for Female** | | |
| --- | --- | --- | --- | --- | --- | --- | --- | --- |
|  |  | **Model 1** | **Model 2** | **Model 3** |  | **Model 1** | **Model 2** | **Model 3** |
| **LAP status** | |  |  |  |  |  |  |  |
|  | Low Lap | 1 (reference) | 1 (reference) | 1 (reference) |  | 1 (reference) | 1 (reference) | 1 (reference) |
|  | High Lap | 1.75(1.20, 2.54)* | 1.45(1.11, 1.89)* | 1.35(1.02, 1.79)* |  | 1.27(0.97, 1.64) | 1.54(1.26, 1.90)* | 1.38(1.10, 1.73)* |
| **Age group** | |  |  |  |  |  |  |  |
|  | 45-49 years | 1 (reference) | 1 (reference) | 1 (reference) |  | 1 (reference) | 1 (reference) | 1 (reference) |
|  | 50-59 years | 1.75(1.20, 2.54)* | 1.73(1.18, 2.53)* | 1.73(1.19, 2.53)* |  | 1.27(0.97, 1.64) | 1.16(0.88, 1.52) | 1.12(0.85, 1.48) |
|  | 60-69 years | 2.11(1.45, 3.07)* | 2.00(1.36, 2.95)* | 2.01(1.36, 2.97)* |  | 1.54(1.19, 2.01)* | 1.36(1.02, 1.81)* | 1.32(0.99, 1.76) |
|  | ≥70 years | 2.00(1.31, 3.06)* | 1.96(1.25, 3.08)* | 2.02(1.28, 3.18)* |  | 1.47(1.07, 2.02)* | 1.20(0.83, 1.74) | 1.17(0.80, 1.71) |
| **Education** | |  |  |  |  |  |  |  |
|  | Illiterate | NA | 1 (reference) | 1 (reference) |  | NA | 1 (reference) | 1 (reference) |
|  | Literate | NA | 1.00(0.67, 1.47) | 0.99(0.67, 1.46) |  | NA | 1.03(0.81, 1.31) | 1.03(0.81, 1.31) |
|  | Primary education | NA | 1.16(0.80, 1.67) | 1.15(0.79, 1.66) |  | NA | 0.73(0.55, 0.96)* | 0.73(0.56, 0.97)* |
|  | Middle school | NA | 0.95(0.65, 1.39) | 0.95(0.65, 1.40) |  | NA | 0.76(0.58, 1.01) | 0.76(0.57, 1.00)* |
| **Marital status** | |  |  |  |  |  |  |  |
|  | Married or cohabiting | NA | 1 (reference) | 1 (reference) |  | NA | 1 (reference) | 1 (reference) |
|  | Single | NA | 0.95(0.64, 1.41) | 0.96(0.65, 1.42) |  | NA | 1.15(0.88, 1.50) | 1.17(0.89, 1.52) |
| **Ln(PCE) by setting** | | |  |  |  |  |  |  |
|  | Bottom tertile | NA | 1 (reference) | 1 (reference) |  | NA | 1 (reference) | 1 (reference) |
|  | Middle tertile | NA | 1.05(0.79, 1.40) | 1.05(0.79, 1.40) |  | NA | 0.94(0.75, 1.17) | 0.94(0.75, 1.18) |
|  | Top tertile | NA | 1.05(0.78, 1.41) | 1.05(0.78, 1.42) |  | NA | 0.94(0.74, 1.19) | 0.94(0.74, 1.19) |
| **Region** | |  |  |  |  |  |  |  |
|  | North China | NA | 1 (reference) | 1 (reference) |  | NA | 1 (reference) | 1 (reference) |
|  | Northeast China | NA | 0.60(0.34, 1.08) | 0.60(0.33, 1.07) |  | NA | 0.52(0.31, 0.86)* | 0.51(0.31, 0.85)* |
|  | East China | NA | 0.79(0.55, 1.14) | 0.79(0.55, 1.14) |  | NA | 0.83(0.61, 1.12) | 0.82(0.60, 1.12) |
|  | Southcentral China | NA | 0.79(0.54, 1.16) | 0.79(0.54, 1.16) |  | NA | 0.94(0.69, 1.29) | 0.93(0.68, 1.28) |
|  | Southwest China | NA | 0.67(0.43, 1.02) | 0.67(0.43, 1.03) |  | NA | 0.81(0.57, 1.14) | 0.81(0.57, 1.16) |
|  | Northwest China | NA | 1.02(0.62, 1.66) | 1.02(0.62, 1.67) |  | NA | 0.78(0.51, 1.19) | 0.79(0.51, 1.21) |
| **Hypertension** | |  |  |  |  |  |  |  |
|  | Never | NA | 1 (reference) | 1 (reference) |  | NA | 1 (reference) | 1 (reference) |
|  | Hypertension | NA | 1.32(1.06, 1.65)* | 1.32(1.05, 1.64)* |  | NA | 1.27(1.05, 1.53)* | 1.27(1.05,1.53)* |
| **Obesity** | |  |  |  |  |  |  |  |
|  | Normal | NA | 1 (reference) | 1 (reference) |  | NA | 1 (reference) | 1 (reference) |
|  | Overweight | NA | 1.19(0.91, 1.56) | 1.18(0.90, 1.55) |  | NA | 1.42(1.13, 1.77)* | 1.40(1.12, 1.76)* |
|  | Obesity | NA | 1.76(1.20, 2.57)* | 1.72(1.17, 2.52)* |  | NA | 1.73(1.30, 2.30)* | 1.71(1.28, 2.27)* |
| **Smoking** | |  |  |  |  |  |  |  |
|  | No Smoking | NA | 1 (reference) | 1 (reference) |  | NA | 1 (reference) | 1 (reference) |
|  | Smoking | NA | 1.19(0.92, 1.53) | 1.18(0.91, 1.52) |  | NA | 1.05(0.74, 1.49) | 1.03(0.72, 1.47) |
| **Drinking** | |  |  |  |  |  |  |  |
|  | No drinking | NA | 1 (reference) | 1 (reference) |  | NA | 1 (reference) | 1 (reference) |
|  | drinking | NA | 1.03(0.82, 1.28) | 1.03(0.82, 1.28) |  | NA | 0.97(0.71, 1.32) | 0.98(0.71, 1.33) |
| **TC** | |  |  |  |  |  |  |  |
|  | ≤200mg/dL | NA | NA | 1 (reference) |  | NA | NA | 1 (reference) |
|  | >200mg/dL | NA | NA | 1.35(1.06, 1.72)* |  | NA | NA | 1.12(0.92, 1.37) |
| **LDL** | |  |  |  |  |  |  |  |
|  | ≤100mg/dL | NA | NA | 1 (reference) |  | NA | NA | 1 (reference) |
|  | >100mg/dL | NA | NA | 0.85(0.67, 1.06) |  | NA | NA | 1.08(0.88, 1.32) |
| **HDL** | |  |  |  |  |  |  |  |
|  | ≥50mg/dL | NA | NA | 1 (reference) |  | NA | NA | 1 (reference) |
|  | <50mg/dL | NA | NA | 1.16(0.91, 1.49) |  | NA | NA | 1.26(1.03, 1.55) |

*Note: All analyses were based on multiply imputed data sets. Data were presented as hazard ratios (95% CI); Associations between LAP and T2DM were conducted using multivariable Cox frailty models with random effect to account for clustering of participants in each province; T2DM, type 2 diabetes mellitus; LAP, lipid accumulation product; PCE, per capita expenditures; TC, total cholesterol; HDL-C, high-density lipoprotein cholesterol; LDL-C, low-density lipoprotein cholesterol; TG, triglyceride;* *NA, not available; * , P<0.05.*

*Model 1 was adjusted for age. Model 2 was adjusted for education, marital status, Ln(PCE) by setting, region, hypertension, smoking, drinking, and general obesity based on Model 1. Model 3 was adjusted for TC, LDL-C and HDL-C based on Model 2.*

**Supplementary Table S7.** Risk of new-onset T2DM by LAP transition in middle-aged and older Chinese based on multiply imputed datasets

| **Model** | **LAP Transitions** | | | |
| --- | --- | --- | --- | --- |
|  | **Group A** | **Group B** | **Group C** | **Group D** |
| Male | 1 (reference) | 1.14 (0.75, 1.74) | 1.29 (0.80, 2.08) | 1.43 (0.99, 2.08) |
| Rural | 1 (reference) | 1.26 (0.80, 1.99) | 1.21 (0.70, 2.08) | 1.41 (0.93, 2.15) |
| Urban | 1 (reference) | 0.55 (0.18, 1.75) | 1.60 (0.56, 4.59) | 1.65 (0.74, 3.70) |
| Female | 1 (reference) | 1.94 (1.42, 2.64)* | 1.38 (0.88, 2.16) | 2.06 (1.50, 2.84)* |
| Rural | 1 (reference) | 1.96 (1.36, 2.83)* | 1.31 (0.75, 2.28) | 2.27 (1.55, 3.32)* |
| Urban | 1 (reference) | 2.01 (1.11, 3.65)* | 1.62 (0.73, 3.57) | 1.84 (1.00, 3.40)* |

*Note: All analyses were based on multiply imputed data sets; Data were presented as hazard ratios (95% CI), adjusted for age, sex, education, region, hypertension, smoking, drinking, TC and LDL-C level; Hazard ratios for T2DM by LAP transitions were calculated using multivariable Cox frailty models with random effect, by which means clustering of participants was accounted for; *P<0.05.*

*The definition from group A to D were listed as following:*

*Group A, maintain Low LAP during follow-up;*

*Group B, Low LAP at baseline turned to High LAP at follow-up;*

*Group C, High LAP at baseline turned to Low LAP at follow-up;*

*Group D, maintain High LAP during follow-up.*
